# Intraoperative imprint cytology versus histological diagnosis for the detection of sentinel lymph nodes in breast cancer treated with neoadjuvant chemotherapy

**DOI:** 10.6061/clinics/2018/e363

**Published:** 2018-07-10

**Authors:** Ronald Enrique Delgado-Bocanegra, Eduardo Camargo Millen, Cristina Moreira do Nascimento, Karine de Aguiar Bruno

**Affiliations:** Instituto Nacional do Cancer (INCA), Rio de Janeiro, RJ, BR

**Keywords:** Imprint, Breast Cancer, Neoadjuvant Chemotherapy, Histology, Accuracy

## Abstract

**OBJECTIVES:**

To compare imprint cytology and paraffin section histology for sentinel lymph node detection in women with breast cancer treated with neoadjuvant chemotherapy.

**METHOD:**

A cross-sectional study and report of the sentinel lymph node statuses of 64 patients with breast cancer who underwent intraoperative imprint cytology and neoadjuvant chemotherapy in a referral cancer institute in Rio de Janeiro, Brazil, between 2014 and 2016.

**RESULTS:**

The mean age was 51 years. The most common histological type was invasive ductal carcinoma (93.75%), and the most common differentiation grade was 2 (62.5%). Overall, 153 lymph nodes were identified, with a mean of 2.39/case. Thirty-four lymph nodes tested positive for malignancy by imprint cytology, and 55 tested positive by histology. Of the 55 positive lymph nodes, 41 (74.5%) involved macrometastases, and 14 (25.5%) involved micrometastases. There were 21 false negatives with imprint cytology, namely, 7 for macrometastases and 14 for micrometastases, resulting in a rate of 17.6%. The sensitivity of imprint cytology was 61.8%, with a specificity and positive predictive value of 100%, a negative predictive value of 82.4% and an accuracy of 86.3%. The method presented null sensitivity for the identification of micrometastases.

**CONCLUSIONS:**

The false-negative rate with imprint cytology was associated with the number of sentinel lymph nodes obtained. The rate found for complete response to neoadjuvant chemotherapy was comparable to the rates reported in the literature. The accuracy of imprint cytology was good, and its specificity was excellent for sentinel lymph node detection; however, the method was unable to detect lymph node micrometastases.

## INTRODUCTION

Breast cancer is the most prevalent cancer type in Brazilian women. According to the National Cancer Institute (INCA), 57,960 new cases are expected for the 2016-2017 period 1.

Axillary staging is a key step in the treatment of breast cancer. For many years, axillary dissection was the standard method used in most cases, being considered an important factor in predicting recurrence and survival 2. In Brazil, more extensive surgeries and axillary dissections are often used, although these procedures are associated with the occurrence of both short- and long-term complications, the most common being hemorrhage, infection, seroma, upper limb lymphedema, chronic pain, and paresthesia resulting from injury to the intercostobrachial nerve 3,4.

Sentinel lymph node biopsy (SLNB) permits lymph node staging and is considered the gold standard method for the evaluation of axillary lymph node status for patients without clinical suspicion of lymph node involvement. SLNB has been used in clinical practice for over a decade and has considerably reduced the morbidity associated with axillary surgery and resulted in axillary lymph node dissection. Now, SLNB is restricted to specific situations 5.

The most relevant studies, including the Axillary Lymphatic Mapping Against Nodal Axillary Clearance (ALMANAC) trial, have recommended SLNB as a safe and effective procedure, with the potential for less morbidity and better quality of life than routine axillary dissection 6. The National Surgical Adjuvant Breast and Bowel Project (NSABP) B-32 trial evaluated 3,986 cases that were divided into two groups, with Group A comprising patients subjected to SLNB followed by axillary lymphadenectomy and with Group B comprising patients subjected to SLNB and subsequent lymphadenectomy alone if the result of the former was positive. No significant differences were found between the two groups with respect to overall survival, disease-free survival or recurrence control. Approximately 85% of cases in both groups underwent adjuvant therapy 7.

A debate has arisen regarding the sentinel lymph node (SLN) identification rate, the false-negative rate, and the accuracy and safety of SLNB when performed after neoadjuvant chemotherapy. Boughey et al. evaluated the SLN identification rate after neoadjuvant chemotherapy in patients enrolled in the American College of Surgical Oncology Group (ACOSOG) Z1071 trial 8. In this study, SLNs were identified in 78.6% of cases when patent blue dye alone was used, in 91.4% of cases when radiolabeled colloid alone was used, and in 93.8% of cases when dual mapping agents were used. A false-negative rate of 12.6% was found when fewer than two SLNs were removed; however, this rate decreased to 9.1% when more than two SLNs were available. Other factors, namely, body mass index, the tumor stage and the patient’s response to neoadjuvant chemotherapy, did not affect SLN detection.

During surgery, pathological analysis is performed on SLNs using techniques that include intraoperative frozen sections, imprint cytology and immunohistochemistry. However, the gold standard method is paraffin section histology following surgery 9.

Of the different methods used to evaluate SLNs, imprint cytology is the most commonly used. The initial route of breast cancer dissemination follows the axillary lymph nodes, and metastatic involvement of the axilla generally progresses from the first to the second and then third level of axillary nodes. Metastases that fail to adhere to this sequence are rare, making up 2% of cases, and are referred to as skip metastases. Therefore, an intraoperative analysis of SLNs that is fast and precise is of vital importance for minimizing the need for repeat surgery in patients with sentinel lymph nodes affected by metastases 10.

In recent years, the use of SLNB has been established as an important tool in the treatment of patients with early stage breast cancer, differentiating those who need radical axillary lymphadenectomy from those who do not. The use of radical axillary lymphadenectomy has declined following the introduction of SLN analysis, with a consequent decrease in the rate of associated complications. Nevertheless, the major concern with SLNB is the occurrence of false negatives from the intraoperative evaluation. False negativity may require a patient to be subjected to an additional surgical procedure, and the proportion of such cases ranges from 4.7 to 16.7% following histological analysis of paraffin-embedded specimens, thus resulting in therapeutic delay, with a significant increase in treatment cost 11-13.

The objective of the present study was to compare imprint cytology with paraffin section histology for the detection of SLNs in women with breast cancer treated with neoadjuvant chemotherapy.

## MATERIALS AND METHODS

This cross-sectional, descriptive study was conducted between 2014 and 2016 and included female patients of any age with a diagnosis of breast cancer and a report of the SLN status. All patients included in this study had undergone complete neoadjuvant chemotherapy at the National Cancer Institute and had been examined by intraoperative imprint cytology. Lymph nodes were identified during surgery using dual mapping agents with radiolabeled colloid and patent blue dye.

The following data were collected from the patients’ charts: age; tumor diameter; the histological characteristics of the tumor (type and grade of differentiation); SLN status based on the intraoperative diagnosis and on the definitive histological diagnosis; and the patient’s clinical and radiological response to chemotherapy, classified as partial or complete. Pathologists at the institute performed intraoperative analysis of the SLNs using imprint cytology, with the slides being observed under an optical microscope. Regardless of the imprint cytology result, the remaining material was paraffin-embedded for the definitive histological diagnosis, thus confirming the positivity or negativity of the lymph node evaluated. Nodal metastases were classified as micrometastases (≤2 mm) or macrometastases (>2 mm) 14.

The sensitivity, specificity, accuracy, and positive and negative predictive values were calculated according to the standard formulae.

### Ethics

This study was approved by the internal review board of the Brazilian National Cancer Institute (INCA), Rio de Janeiro, Brazil. The procedures were in accordance with the ethical standards of the responsible committee and with the Helsinki Declaration of 1975, as revised in 1983. The principal investigator alone collected all of the data, which were immediately anonymized.

## RESULTS

All medical charts available for the study period were reviewed according to the inclusion criteria, resulting in 64 charts being selected. The mean age of the patients was 51 years (range 25-83 years). Thirty-two patients (50%) were ≤50 years of age, and 44 women (68.8%) were menopausal. The most common histological type was invasive ductal carcinoma, making up 93.75% of cases, with the remaining cases comprising invasive lobular carcinoma. The most common grade of differentiation was 2 (62.5%), followed by 3 (26.2%) and 1 (10.9%). Immunohistochemistry revealed that 14% of the cases were triple-negative, 15.6% were human epidermal growth factor receptor 2 (HER2)-positive, and 73% were estrogen receptor (ER)-positive. Regarding tumor size, 51.5% of the cases were classified as T2, namely, between 2 and 5 cm, 35.9% were classified as T3, and 12.5% were classified as T1. No lymph node involvement was present in 62.5% of the cases; however, 32.8% were classified as N1, and 4.7% were classified as N2 (Table 1).

A total of 153 lymph nodes were identified in the 64 cases, with a mean of 2.39 lymph nodes per patient. A total of 34 lymph nodes tested positive for malignancy by intraoperative imprint cytology. Later, definitive histological diagnoses using paraffin-embedded surgical specimens revealed a total of 55 positive lymph nodes (Table 2).

These 55 positive lymph nodes were subdivided into 41 positive cases involving macrometastases and 14 positive cases involving micrometastases. Imprint cytology yielded 21 false negatives: 7 referring to macrometastases (Table 3) and 14 referring to micrometastases (Table 4). The false-negative rate was 17.6% for imprint cytology.

In 24 cases, the clinical and radiological response to neoadjuvant chemotherapy was complete. In the cases with false negatives for macrometastases, the response to chemotherapy was partial, while in 4 cases (28.6%) with false negatives for micrometastases, the response to chemotherapy was complete.

Overall, when both macrometastases and micrometastases were considered, imprint cytology had a sensitivity of 61.8%, a specificity and positive predictive value of 100%, a negative predictive value of 82.4%, and an accuracy of 86.3%. When stratified into micrometastases and macrometastases, sensitivity was null for micrometastases, so specificity was 100% (Table 5).

## DISCUSSION

Breast cancer management has undergone many changes in recent years, particularly where treatment is concerned, with the objective of improving the effectiveness and tolerance of treatment. Axillary status is an important prognostic factor in breast cancer and may be a determinant indicating whether systemic adjuvant therapy should be administered 12. Axillary management in patients with breast cancer has improved significantly over recent decades, with SLNB having become the standard procedure for axillary staging in patients without clinically detectable lymph nodes (cN0) 11.

The National Comprehensive Cancer Network (NCCN) and ACOSOG have proposed that axillary lymphadenectomy be restricted to specific situations, based on the failure of the ACOSOG Z0011 trial to find any differences in disease-free survival or overall survival between the groups randomized to axillary lymphadenectomy and SLNB alone groups 15. Accordingly, axillary lymphadenectomy could be ruled out for patients subjected to conservative surgery, those with T1-T2 tumors, those with ≤2 positive SLNs, those undergoing radiotherapy planning, and those not subjected to neoadjuvant chemotherapy. These recommendations have led to a reduction in the rate of axillary lymphadenectomies in breast cancer patients with positive SLNBs worldwide 16.

Additionally, according to the NCCN, patients suitable for neoadjuvant chemotherapy with no axillary involvement detected by clinical palpation or ultrasound prior to initiating chemotherapy are candidates for SLNB following treatment. If any suspicious ultrasound images exist, fine-needle aspiration (FNA) biopsy should be performed. If no lymph node involvement is detected, SLNB can be performed after neoadjuvant chemotherapy. On the other hand, if the FNA biopsy is positive prior to chemotherapy, axillary restaging should be performed after chemotherapy, with axillary lymphadenectomy being indicated if the axilla is clinically affected or with SLNB if the axilla is clinically negative. For these patients, intraoperative evaluations of SLNs should be performed because if the lymph nodes are affected, axillary lymphadenectomy is recommended 15.

Mansel et al. 6 reported data for a group of patients with positive lymph nodes that were identified prior to neoadjuvant chemotherapy. SLNB was shown to have a false-negative rate above 10% after chemotherapy. In the present study, imprint cytology had an overall rate of false-negative lymph nodes (i.e., failing to identify both macrometastases and micrometastases) of 17.6% compared with paraffin section histology.

Neoadjuvant chemotherapy is a widely accepted treatment for breast cancer, and neoadjuvant chemotherapy and adjuvant therapy are equally effective options. The principal advantages include the possibility of conducting an *in vivo* evaluation of the sensitivity of the tumor to chemotherapy, the likelihood of reducing micrometastatic disease and the consequent need for a less extensive form of surgery with less morbidity 17.

Recently, the early detection rate, better known as downstaging, has been approximately 94% in patients with breast cancer. Furthermore, a pathological complete response has been achieved in 20-40% of patients following neoadjuvant chemotherapy. Evidently, a pathological complete response is associated with a better prognosis and longer overall survival 18. Neoadjuvant chemotherapy can decrease the risk of residual axillary disease and may be able to completely eradicate axillary metastases detected by histology in patients with locally advanced breast cancer 19.

The SENTINA study showed that lymph node disease was restricted to the SLNs in 58% of patients who converted from clinically node-positive to clinically node-negative breast cancer following neoadjuvant chemotherapy, with the false-negative rate decreasing significantly with the number of SLNs removed. In patients who had one SLN removed, the false-negative rate was 24.3% compared to 18.5% when two SLNs were removed and less than 10% when three SLNs were removed. Another important finding was that the false-negative rate was 8.6% in patients subjected to dual mapping agents (radiolabeled colloid and patent blue) for the detection of SLNs 20. In the present study, dual mapping agents had been used in all selected cases, and the mean number of SLNs obtained per case was 2.39. Since there was no axillary dissection in the cases that tested negative according to imprint cytology and histology, calculating the false-negative rate of SLNB was impossible in the present study. The false-negative rate with imprint cytology was 17.6%, and in most cases, false negativity resulted from the presence of micrometastases.

The ACOSOG Z1071 trial also reported that axillary involvement became clinically undetectable in 83% of patients evaluated after neoadjuvant chemotherapy and in 12% of those with palpable nodules. In the women with breast cancer with cN1 disease who received neoadjuvant chemotherapy and who had two or more SLNs examined, the false-negative rate exceeded 10%, which is considered acceptable. Given this threshold of acceptability, changes in the management and selection of patients to achieve greater sensitivity are needed before SLNB can be accepted as an alternative to axillary lymphadenectomy in this patient population 21.

Currently, intraoperative evaluations are performed by imprint cytology, frozen section examination or both. Although immunohistochemistry and intraoperative molecular techniques are also available, they are not commonly used 22.

Intraoperative imprint cytology is comparable to frozen section examination for the rapid evaluation of the SLNB, with the advantages of speed, reliability, reduced cost and better tissue preservation 23,24. Furthermore, the surgeon is alerted to the presence of a lymph node metastasis, thus reducing the number of repeat axillary lymphadenectomy surgeries, particularly in patients subjected to simple mastectomy. This technique also allows patients and their families to be informed immediately regarding lymph node status 23.

The routine use of SLNB for the evaluation of residual lymph node metastasis after neoadjuvant chemotherapy has not been recommended for patients who initially tested positive for malignancy. In these cases, axillary management after neoadjuvant chemotherapy should be individualized in accordance with the clinical subtype of the tumor, with SLNB being performed in selected patients whenever possible 25.

Another study comparing imprint cytology with the histological analysis of SLNs from patients with locally advanced breast cancer subjected to neoadjuvant chemotherapy concluded that imprint cytology could not be recommended given the high rate of false negatives (12.01%) observed in the study. Therefore, further studies are required to validate the method 26.

In this study, the accuracy of imprint cytology, particularly for the detection of macrometastases, was good. In addition, specificity was excellent, namely, no negative lymph nodes were identified as positive. Overall, the sensitivity of the technique was poor for the detection of both macrometastases and micrometastases. Although the method failed to identify micrometastases, as reflected in the null sensitivity, the sensitivity for the detection of macrometastases alone was 83%. In this respect, there are indeed some limitations with imprint cytology in the identification of micrometastases, which can be detected by subsequent histology or immunohistochemistry 27.

With the objective of comparing imprint cytology to histology for the evaluation of SLNs in 34 patients subjected to neoadjuvant chemotherapy, Aguiar 28 reported a sensitivity of 90.9%, with a false-negative rate of 7.46% and accuracy of 95.7%. Those results differ from the findings of the present study in that the sensitivity in the present study was lower (61.8%), and the false-negative rate in the present study was 17.6%. Nevertheless, the accuracy here was also good (86.3%).

A study conducted by Dedivitis et al. 29 compared the analysis of neck nodes by imprint cytology and FNA biopsy in 86 patients and reported similar results, with a sensitivity of 93.5%; a specificity of 100%; negative and positive predictive values of 85.7% and 100%, respectively; and an accuracy of 95.3%. Therefore, in view of the high accuracy associated with imprint cytology, these authors suggested its use as an additional aid to histology.

Intraoperative SLNB assessment by imprint cytology has relied on the fact that patients may be spared a second surgery when the SLN is positive 30. According to the study by Tew K, et al., intraoperative imprint cytology has a sensitivity of 63% with a false-negative rate of 37%, and the pooled sensitivity for macrometastases is higher (81%) than that for micrometastases (22%) 31. Our study showed a false-negative rate of 17.6% for imprint cytology and a sensitivity of 0% for micrometastases. In addition, since the publication of the Z0011 trial (where patients with 1 or 2 positive SLNs did not benefit from a complete axillary lymphadenectomy), the use of intraoperative assessment of the SLN in the adjuvant setting has decreased 32. However, in the neoadjuvant chemotherapy setting, intraoperative assessments of SLNs are still considered necessary. The standard procedure for positive SLNs after neoadjuvant chemotherapy is axillary lymphadenectomy, so patients with positive SLNs intraoperatively may undergo axillary lymphadenectomy within the same surgery, avoiding the drawbacks of a second surgery 33. For this reason, patients in the neoadjuvant chemotherapy setting with intraoperative false-negative imprint cytology results will require a second surgery.

SLNB provides important information on lymph node staging and reduces the morbidity associated with unnecessary axillary lymphadenectomy for patients with breast cancer and no clinically detectable lymph node involvement. The false-negative rate with imprint cytology, which is associated with the number of lymph nodes removed, and the complete response rate to neoadjuvant chemotherapy currently achieved are comparable to the rates published in the literature. The accuracy of imprint cytology is good, and its specificity is excellent for the detection of SLNs; however, it fails to detect lymph node micrometastases, and this is important because all patients with intraoperative imprint cytology in the neoadjuvant chemotherapy setting who have false-negative results, will require a second surgery.

Performing SLNB after neoadjuvant chemotherapy may result in a high rate of false negatives, a finding that has already been reported in several studies. Therefore, studies such as this one, which are designed to compare methods for the intraoperative analysis of SLNs with a definitive histological diagnosis of a paraffin-embedded portion that is analyzed by hematoxylin-eosin staining, are of utmost importance.

## AUTHOR CONTRIBUTIONS

Delgado-Bocanegra RE and Millen EC contributed to the conception of the research. Delgado-Bocanegra RE contributed to the design of the research. Delgado-Bocanegra RE and Bruno KA contributed to the acquisition and analysis of the data. Delgado-Bocanegra RE, Millen EC, Nascimento CM and Bruno KA contributed to the interpretation of the data. Delgado-Bocanegra RE and Bruno KA drafted the manuscript. All authors have critically revised the manuscript, agreed to be fully accountable for ensuring the integrity and accuracy of the work, and read and approved the final version of the manuscript.

## Figures and Tables

**Table 1:** Baseline clinical characteristics of the study sample (n=64).

	n (%)
**Age group (years)**	
<30	2 (3.2)
30–40	12 (18.7)
41–50	18 (28.1)
>50	32 (50)
**Histological type**	
Invasive ductal carcinoma	60 (93.75)
Invasive lobular carcinoma	4 (6.25)
**Grade of differentiation**	
Grade 1	7 (10.9)
Grade 2	40 (62.5)
Grade 3	17 (26.6)
**Tumor size**	
T1 (<2 cm)	8 (12.5)
T2 (2-5 cm)	33 (51.6)
T3 (>5 cm)	23 (35.9)
**Lymph node involvement**	
No involvement (N0)	40 (62.5)
N1	21 (32.8)
N2	3 (4.7)
**Immunohistochemistry**	
Triple-negative	9 (14)
HER2-positive	10 (15.6)
HER2-negative	54 (84.4)
Estrogen receptor-positive	47 (73.43)
Estrogen receptor-negative	17 (26.57)
Progesterone receptor-positive	40 (62.5)
Progesterone receptor-negative	24 (37.5)

HER2: Human Epidermal Growth Factor Receptor 2.

**Table 2:** Comparison of imprint cytology and histology for the detection of sentinel lymph node positivity for macrometastases and micrometastases.

	Positive by histology	Negative by histology	Total n (%)
Positive by imprint cytology	34	0	34 (22.2)
Negative by imprint cytology	21	98	119 (77.8)
**Total n (%)**	55 (35.9)	98 (64.1)	**153 (100)**

**Table 3:** Comparison of imprint cytology and histology for the detection of sentinel lymph node positivity for macrometastases.

	Positive by histology	Negative by histology	Totaln (%)
Positive by imprint cytology	34	0	34 (24.5)
Negative by imprint cytology	7	98	105 (75.5)
**Total n (%)**	41 (29.5)	98 (70.5)	**139 (100)**

**Table 4:** Comparison of imprint cytology and histology for the detection of sentinel lymph node positivity for micrometastases.

	Positive by histology	Negative by histology	Total n (%)
Positive by imprint cytology	0	0	0 (0)
Negative by imprint cytology	14	98	112 (100)
**Total n (%)**	14 (12.5)	98 (87.5)	**112 (100)**

**Table 5:** Sensitivity, specificity, positive and negative predictive values, and accuracy of imprint cytology.

Imprint cytology	Sensitivity	Specificity	Positive predictive value	Negative predictive value	Accuracy
Lymph node positivity for macrometastases and micrometastases	61.8%	100%	100%	82.4%	86.3%
Lymph node positivity for macrometastases	82.9%	100%	100%	93.3%	95%
Lymph node positivity for micrometastases	0%	100%	0%	87.5%	87.5%
